# A plasmid toolkit for cloning chimeric cDNAs encoding customized fusion proteins into any Gateway destination expression vector

**DOI:** 10.1186/1471-2199-14-18

**Published:** 2013-08-20

**Authors:** Raquel Buj, Noa Iglesias, Anna M Planas, Tomàs Santalucía

**Affiliations:** 1 Department of Brain Ischemia and Neurodegeneration, Institut d’Investigacions Biomèdiques de Barcelona (IIBB)-Consejo Superior de Investigaciones Científicas (CSIC), Barcelona, Spain; 2 Institut d’Investigacions Biomèdiques August Pi i Sunyer (IDIBAPS), Barcelona, Spain; 3 Institut de Medicina Predictiva i Personalitzada del Càncer (IMPPC), Badalona, Barcelona, Spain

**Keywords:** Gateway cloning, Fusion protein, Combinatorial, Fluorescent protein, Epitope tag

## Abstract

**Background:**

Valuable clone collections encoding the complete ORFeomes for some model organisms have been constructed following the completion of their genome sequencing projects. These libraries are based on Gateway cloning technology, which facilitates the study of protein function by simplifying the subcloning of open reading frames (ORF) into any suitable destination vector. The expression of proteins of interest as fusions with functional modules is a frequent approach in their initial functional characterization. A limited number of Gateway destination expression vectors allow the construction of fusion proteins from ORFeome-derived sequences, but they are restricted to the possibilities offered by their inbuilt functional modules and their pre-defined model organism-specificity. Thus, the availability of cloning systems that overcome these limitations would be highly advantageous.

**Results:**

We present a versatile cloning toolkit for constructing fully-customizable three-part fusion proteins based on the MultiSite Gateway cloning system. The fusion protein components are encoded in the three plasmids integral to the kit. These can recombine with any purposely-engineered destination vector that uses a heterologous promoter external to the Gateway cassette, leading to the in-frame cloning of an ORF of interest flanked by two functional modules. In contrast to previous systems, a third part becomes available for peptide-encoding as it no longer needs to contain a promoter, resulting in an increased number of possible fusion combinations. We have constructed the kit’s component plasmids and demonstrate its functionality by providing proof-of-principle data on the expression of prototype fluorescent fusions in transiently-transfected cells.

**Conclusions:**

We have developed a toolkit for creating fusion proteins with customized N- and C-term modules from Gateway entry clones encoding ORFs of interest. Importantly, our method allows entry clones obtained from ORFeome collections to be used without prior modifications. Using this technology, any existing Gateway destination expression vector with its model-specific properties could be easily adapted for expressing fusion proteins.

## Background

The availability of genomic data from an ever increasing number of species, arising from the completion of multiple genome projects and the metagenomic analysis of natural samples, has uncovered a potential treasure trove of proteins with as-yet undiscovered properties that could be exploited to solve both existing and future biotechnology challenges. The huge amount of sequence data currently being generated calls not only for bioinformatic prediction of open-reading frames (ORFs) and their putative functions [1], but also for novel screening techniques (functional metagenomics) that will help identifying gene products with potential technological interest [2]. Metagenomics is already revealing a wealth of new protein families with unknown functions, as well as distant relatives of other proteins with well-characterised functions, which will be very valuable for evolutionary and structural studies [3]. These studies will likely require the expression of newly-described ORFs in heterologous hosts for their initial characterisation, and expressing them as protein fusions with standard tags might be advantageous. One of the main hurdles in metagenomic screening derives from the existence of profound differences in the way that different taxonomic groups of organisms translate ORFs (i.e. codon bias and use of different initiation codons) [2]. These differential features demand for the creation of flexible cloning and expression systems allowing protein expression in multiple hosts [4]. Furthermore, the prediction of the whole ORF complement of an organism (the so called ORFeome) based on the analysis of genomic data has facilitated the creation of representative clone collections that contain a large proportion of such ORFs [5]. The functional characterization of putative ortholog genes identified among ORFeome clone collections might involve monitoring their intracellular behaviour, a task facilitated by the generation of fusions with genetically-encoded fluorescent proteins [6]. Controlling for possible artefacts caused by steric interference of the fluorescent module with important functional domains in the protein of interest should ideally involve comparing the intracellular behaviour when the fluorescent module is attached to either the N- or the C-termini, as well as with that of the protein over-expressed on its own [7], as it may explain unexpected behaviours. It might also be necessary to exchange the fluorescent module so to find the one with the spectral properties best suited to a particular experiment [7]. Meeting those demands may lead to time-consuming subcloning strategies, which would be further complicated by the intrinsically limited availability of in frame-, pre-inserted functional modules in expression vector backbones. The simplification of subcloning strategies, at least allowing the simultaneous insertion of the ORFs of interest into a panel of different expression vectors, would be advantageous [5]. One crucial step in this direction has been the implementation of recombination-based cloning systems such as the Gateway kits commercialized by Invitrogen (Life Technologies). Gateway cloning, which has been adopted for the creation of many ORFeome clone collections [5], bypasses the constraints imposed by restriction and ligation strategies and, in its basic configuration, is based on the recombinase-mediated shuttling of a DNA fragment (e.g. an ORF) from an entry clone into a destination vector. DNA fragments are transferred between vectors regardless of their sequence thanks to flanking recognition signals (the *att* sites) targeted by recombinases that have been borrowed from the λ phage’s lysogeny cycle machinery [8]. The creation of an entry clone typically (but not exclusively) involves the PCR-mediated attachment of flanking *attB* sites to the DNA of interest, and its recombination-mediated cloning into an *attP*-containing plasmid. Recombination of this entry clone with an appropriate destination vector containing compatible *att* sequences will lead to an expression vector encoding an ORF, provided that the destination vector is furnished with a suitable promoter. The expression of an ORF of interest as a fusion with a fluorescent protein can be achieved with the help of a limited number of commercially-available destination vectors that contain an in-frame pre-inserted module.

Our aim was to develop a system that could meet the above requirements while avoiding complex subcloning strategies. Owing to the advantages of Gateway cloning, we generated a customizable Gateway-based plasmid toolkit for constructing fusion proteins from any ORF available as a standard entry clone. ORFs can be optionally expressed on their own or as in-frame fusions with a collection of standardized functional modules, in a combinatorial way, and from any Gateway destination expression vector. We describe the design and components of the toolkit, and we demonstrate its feasibility by presenting proof-of-principle expression data obtained in transient transfection experiments with prototype vectors encoding fluorescent fusion proteins*.*

## Results and discussion

### Conceptual design of the cloning toolkit

**Figure 1:**
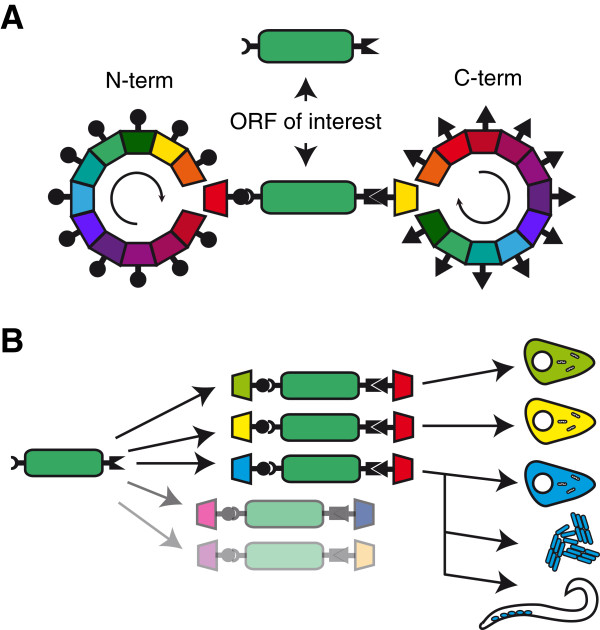
**A combinatorial strategy for the expression of proteins as fusions with functional modules. A**. Our toolkit’s design allows any ORF of interest to be easily expressed either on its own (above) or as a fusion (below) with N- and C-terminal modules picked from among a collection of parts (represented as multicoloured wheels). The fusion’s components are linked by standardized short peptide arms (black spheres and triangles) introduced as a result of the cloning procedure. The collection is constructed so that a given functional module can be attached either at the N-terminal or the C-terminal side of the protein of interest, which is helpful for finding the optimal arrangement in each case. **B**. Multiple fusions can potentially be generated by combining an ORF of interest with different modules from the collection. Specific fusions aimed at particular applications will be defined by the choice of modules used. For example, an ORF of interest N-terminally tagged with different fluorescent modules (green, yellow, blue) can be used to monitor the fusion protein’s intracellular localization under conditions best suited for each experiment. Furthermore, our versatile design allows a particular fusion protein arrangement to be cloned into vectors specific for expression in different model systems in the context of comparative studies.

**Figure 2:**
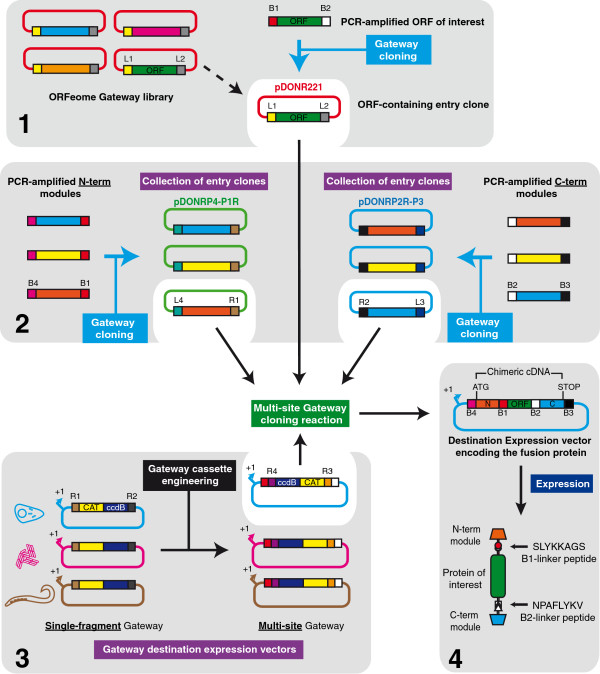
**Strategy for the cloning of fusion proteins.** Cloning is based on the Multi-site Gateway kit from Invitrogen. The plasmid encoding the ORF of interest can either be an existing entry clone from a Gateway-based ORFeome library, or can be constructed by Gateway-cloning the PCR-amplified ORF into pDONR221 with BP clonase **(panel 1)**. A collection of entry clones encoding the functional modules to be attached to the ORF of interest is constructed similarly by Gateway cloning the PCR-amplified modules into the pDONR P4-P1R (N-term modules) and pDONR P2RP3 (C-term modules) plasmids from the MultiSite Gateway kit (**panel 2**, see main text for details). All of the inserts in the entry vectors are flanked by appropriate *att* sites provided by the PCR-amplification primers, and are indicated throughout this figure with a key (e.g. the *attB1* site is indicated as *B1*). Single-fragment Gateway destination expression vectors designed for expression in different model systems are adapted by engineering of the Gateway cassette to allow MultiSite Gateway cloning reactions **(panel 3)**. Such reactions involve one adapted destination vector, as well as the plasmids encoding the ORF of interest and the chosen N-term and C-term modules (white background), whose matching *att* sites (R4xL4, R1xL1, L2xR2, L3xR3) recombine to produce a destination expression vector encoding the fusion protein in the form of a chimeric cDNA **(panel 4)**. Expression of the cDNA results in the production of a fusion protein whose three constituent parts are linked by short peptides resulting from the translation of the *attB1* and *attB2* sites remaining after the LR recombination (panel 4, see main text for details).

The construction of a vector for the expression of fusion proteins requires the subcloning of the cDNA of interest into a vector’s backbone, at a site next to one or more pre-existing in-frame modules that provide the functions to be monitored. Hence, the design of the preassembled backbone vector conditions the nature of the fusion and the relative position of its constituent modules. This limits the possibilities for screening the best modular arrangement and performance. Subcloning of fusion proteins could be streamlined by releasing the functional modules from a fixed position in the backbone vector and regarding them as exchangeable modules to be cloned at the N- and C-termini simultaneously with the ORF of interest (Figure 1A). Ideally, such an approach would be based on recombination-mediated cloning since this should allow standardisation of the procedures and of the peptides linking the modules, facilitating the adoption of high-throughput technology, as described in earlier reports [9,10]. Expressing a protein optionally on its own or as a fusion with two flanking modules from a single vector backbone would be an additional asset (Figure 1A). Thus, when expressed under uniform conditions, comparing a protein’s behaviour in either circumstance would facilitate the identification of potential artefacts occurring as a consequence of the fusion with functional modules [6]. It would also allow a large number of fusions based on a given ORF to be cloned simultaneously into an array of model-specific expression vectors in combination with a collection of functional modules (Figure 1B). A cloning toolkit with such features would represent an advance over previous systems constructed according to similar design principles [10]. Furthermore, our toolkit would ideally tap into the wealth of ORFeome resources produced by the efforts of different laboratories leading the mass-production of parts for systems biology [5], a feature shared with previous designs [9,10]. We chose a cloning toolkit developed with the MultiSite Gateway three-fragment vector construction kit from Invitrogen (See Additional file 1) as it would make it meet the requirements above. A recombination reaction involving three separate entry clones based on the pDONR vectors from the kit (pDONRP4-P1R, pDONR221 and pDONRP2R-P3) and containing DNA fragments flanked by specific variants of the *att* sites, results in their ordered subcloning into the kit’s promoterless destination vector pDEST-R4-R3 (See Additional file 1). Interestingly, the DNA fragment that occupies the central position is flanked by *attL1* and *attL2* sites in its entry vector (pDONR221), just as in the entry vectors available from public or commercial cDNA resources (e.g. ORF shuttle clones available through the ORFeome Collaboration [11,12]). Furthermore, new entry clones constructed by PCR-amplification of cDNAs and their BP clonase-mediated recombination into vectors containing *attP1/attP2* sites used for standard Gateway cloning are also compatible with the MultiSite Gateway kit (Figure 2, panel 1) (Also see Additional file 1). The preservation of the translation frame of the three fragments that are pieced together was built into the original MultiSite cloning system’s design [9]. Therefore, by cloning the N-term- and C-term-functional modules proposed in the toolkit’s design into pDONR-P4-P1R and pDONR-P2R-P3, respectively, they could join an entry vector holding the ORF for a protein of interest in a MultiSite cloning reaction that would create a fusion protein’s chimeric ORF (Figure 2, panel 2). However, pDEST-R4-R3 is a promoterless destination vector, meaning that further engineering of this vector would be required in order to insert a promoter upstream of the chimeric ORF. Instead, replacing the original single-fragment Gateway cassette in any destination expression vector furnished with a heterologous promoter, with the R4-R3 cassette from pDEST-R4-R3 seemed a more powerful strategy (Figure 2, panel 3). This would allow taking advantage of numerous existing Gateway destination vectors that would become in this way available for MultiSite Gateway cloning (see also ref. [13]), increasing the functional flexibility of the toolkit. This way, the protein of interest could be expressed on its own or as a fusion protein in the context of the same vector backbone, by choosing for the LR-recombination between the unmodified vector and that with the engineered Gateway cassette, used in combination with the clones encoding functional modules. Furthermore, all of the elements in the proposed collections of flanking N-terminal and C-terminal functional modules would partake of the same construction principles. Thus, they could be used in a combinatorial fashion in MultiSite Gateway LR Clonase-mediated recombination reactions with any existing Gateway entry clone containing an *attL1/attL2*-flanked ORF, where the components of the fusion proteins would be linked by the translated remaining *attB1* and *attB2* sequences acting as universal connectors (Figure 2, panel 4). The open architecture of such toolkit would allow for easily updating any successful fusion arrangement to the latest versions of the functional modules in use (e.g. fluorescent proteins). This would be achieved by simply incorporating such versions as new modules in the collection, so they could be used instead of the superseded modules in a replay of the recombination reaction that led to the fusion in question.

### Engineering of a destination expression vector to enable three-fragment cloning

**Figure 3:**
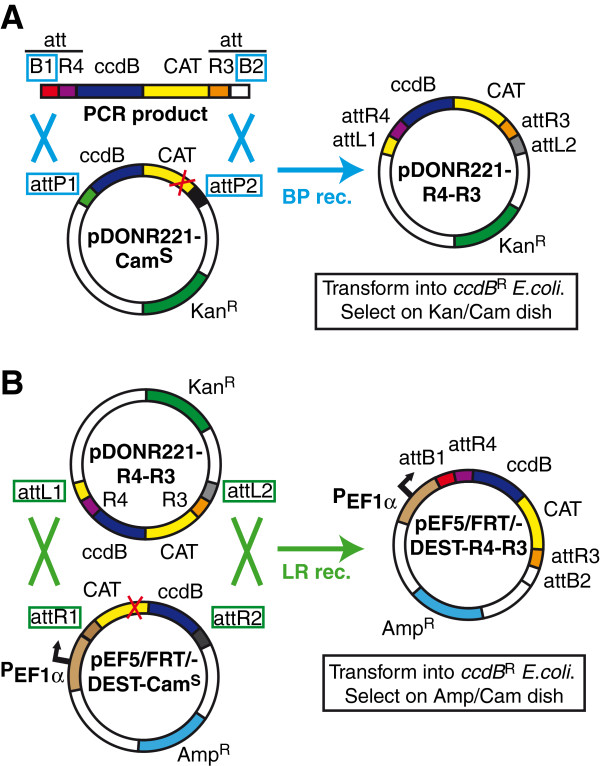
**Adaptation of existing Gateway Destination vectors for the expression of fusion proteins. A**. Construction of adapter vector pDONR221-R4-R3. A PCR product corresponding to the *attR4*-*attR3*-containing Gateway cassette in pDEST-R4-R3 was amplified with primers that provided flanking *attB1* and *attB2* sites. An inactivating mutation was introduced into the *CAT* gene (red X) in pDONR221 by site-directed mutagenesis so that ccdB-resistant *E. coli* transformed with this plasmid were sensitive to chloramphenicol (Cam^S^). A BP-recombination reaction was set up between the amplified *attR4-attR3* Gateway cassette and pDONR221_Cam^S^ to create pDONR221-R4-R3 (see details under methods). The *att* sites involved in the reaction are marked with blue squares. Selection with LB medium containing kanamycin and chloramphenicol (*Kan*/*Cam*) yielded colonies containing the recombined plasmid. **B**. Adaptation of destination expression vector pEF5/FRT/V5-DEST for MultiSite Gateway recombination reactions. The same inactivating mutation in the *CAT* gene as above (red X) was introduced into the commercially available single-site destination expression vector pEF5/FRT/V5-DEST to generate a Cam-sensitive version (pEF5/FRT/DEST-Cam^S^). The mutant plasmid was subjected to an LR-recombination reaction with pDONR221-R4-R3 in order to replace its original Gateway cassette with the *attR4-attR3* MultiSite recombination cassette in pDONR221-R4-R3 (endowed with a wild type *CAT* gene). The *att* sites involved in the reaction are marked with green squares. Selection of the colonies containing recombined pEF5/FRT/V5-DEST (pEF5/FRT/-DEST-R4-R3) was carried out with LB medium containing ampicillin and chloramphenicol (*Amp/Cam*). Only the features in the pEF5/FRT/V5-DEST plasmid map that are relevant for the reaction are shown.

The substitution of the single site- by the MultiSite *attR4-attR3* Gateway cassette in an existing destination expression vector was achieved by modifying a method first reported by Magnani and colleagues [13]. This consists of an LR-recombination reaction between a vector that contains the *attR4-attR3* Gateway cassette from pDEST-R4-R3, flanked by *attL1/attL2* sites (pDONR221-R4-R3), and the destination expression vector in its standard form, furnished with *attR1/attR*2 sites [13]. In this way, a successful recombination reaction would place the R4-R3 Gateway cassette between *attB1* and *attB2* sites (resulting from the LR recombination between *attL1/attL2* and *attR1/attR2* sites). However, such a procedure raised a technical issue, common to all reactions where standard Gateway cassettes are replaced with versions containing different flanking recombination sites, i.e. how to easily distinguish, after bacterial transformation, between *E. coli* colonies that contain the original destination vector from those containing the vector with the recombined R4-R3 Gateway cassette, since they would be identical in terms of their selection markers. To solve this issue, we took advantage of the fact that Gateway vectors typically possess two antibiotic resistance genes, one of them being the chloramphenicol-acetyl transferase (*CAT*) gene that lies inside the Gateway cassette, and another one that lies elsewhere in the vector. The ccdB-resistant *E. coli* bacteria transformed with such vectors acquire resistance to both chloramphenicol and the antibiotic (ampicillin, kanamycin, etc.) specified by the other resistance gene. Our strategy consisted in engineering the vector before the recombination reaction, so that an inactivating mutation was introduced by site-directed mutagenesis into the *CAT* gene in the existing Gateway cassette, while the rest of the vector’s functionality was preserved. *E. coli* cells transformed with this vector were sensitive to the presence of chloramphenicol in the culture medium (See Additional file 2). On the other hand, the PCR-amplified MultiSite Gateway cassette, which was to replace the original one after recombination, had a functional *CAT* gene. Thus, successful recombination would restore the resistance to chloramphenicol in the engineered vector and allow the growth of colonies of bacteria in the presence of chloramphenicol and ampicillin, while negatively selecting bacteria transformed with the non-recombined destination vector. Since all Gateway vectors contain the same sequence in the *CAT* gene that is targeted by our mutagenesis reaction, this strategy could potentially be used for tailoring the Gateway cassette in any vector. We initially tested it by assembling the pDONR221-R4-R3 vector (Figure 3A). This involved a BP-recombination reaction between a PCR-amplified R4-R3 Gateway cassette furnished with a wild-type *CAT* gene, and a *CAT*-mutant version of the pDONR221 vector (Figure 3A). The screening strategy proved to be successful, and we were able to isolate bacterial clones containing recombined pDONR221-R4-R3, as confirmed by sequencing, which were again resistant to chloramphenicol and kanamycin. We subsequently used the same strategy in the adaptation of a destination expression vector for MultiSite Gateway cloning, as required by the design of our cloning toolkit. Thus, an LR-recombination reaction was set up between pDONR221-R4-R3 and the *CAT*-mutant version of pEF5/FRT/V5-DEST (Figure 3B). After transformation of the reaction and plating on LB medium supplemented with ampicillin and chloramphenicol, several colonies resistant to both antibiotics were isolated. Sequencing of the plasmid prepared from these colonies evidenced the presence of an R4-R3 Gateway cassette flanked by *attB1/attB2* sites in the context of pEF5/FRT/V5-DEST, showing the successful substitution of the single site- by the MultiSite Gateway cassette. Thus, we designated this plasmid pEF5/FRT/V5-DEST-R4-R3 and used it in the construction of the fusion protein prototypes discussed below. Importantly, the mutation of the destination vectors’ *CAT* gene required by our method will not only be useful for enabling MultiSite recombination cloning, but could also be used subsequently for other adaptation reactions involving different Gateway cassettes flanked by other types of recombination sites (different *att* sequences, FRT or *loxP* recombination sites), which could be screened with the same procedure.

### Creation of a collection of functional modules

We cloned a collection of functional DNA modules into vectors pDONR-P4-P1R and pDONR-P2R-P3 so they could be fused to the N- or C-termini of the protein of interest, respectively. This was carried out by BP-recombination between the *attB*-flanked PCR products encompassing the functional modules and either of the pDONR vectors. PCR products that were to be cloned into pDONR-P4-P1R were flanked by *attB4/attB1* sites, while those to be cloned into pDONRP2R-P3 were flanked by *attB2/attB3* sites (Figure 2, panel 2). A current list of the components in our collection of modules is provided as Table 1. At the moment, they mostly consist of clones containing the cDNA for a series of fluorescent proteins, which can be fused either at the N-terminal or C-terminal end of the ORF of interest. The fluorescent proteins available are ECFP (cyan), EGFP (green), EYFP (yellow) and mKate2 (far red). There is also a 3′-module that allows expressing ECFP from an internal ribosome entry site (IRES) in the context of a bicistronic mRNA shared with a two-module fusion protein encoded at the 5′-half of the mRNA. We will introduce modules encoding the A206K EGFP and EYFP variants, with reduced oligomer formation [14], to be used in fusions whose behaviour could be affected by fluorescent protein oligomerization. This will not be necessary for ECFP-encoding modules since this protein carries a constitutive mutation that prevents its dimerization [15]. Since the three entry vectors from the MultiSite Gateway cloning kit must be present for successful recombination, we included a non peptide-encoding 3′-module with a stop codon and a SV40 early polyadenylation signal cassette to allow translational termination of chimeric ORFs when fusion of a module at the C-terminus is not necessary or convenient. Please note that the parental pEF5/FRT/V5-DEST vector contains the BGH polyadenylation signal downstream of the Gateway cassette and this is preserved in pEF5/FRT/V5-DEST-R4-R3. Thus, when a 3′ peptide-encoding module is typically used, the BGH polyA signal will allow proper processing of the chimeric transcript, with the 3′ module providing the STOP codon at the end of the encoded peptide. Because an N-terminal tag might interfere with functionally important modifications of the protein (e.g. myristoylation), we will also obtain a neutral 5′-module containing an intronic sequence that can serve a similar function at the N-terminus. We have also constructed modules containing the well-characterised V5 and 6xHis epitope tags. Other possible modules would be those encoding for protein domains that target proteins to an organelle (e.g. NLS and a mitochondrial targeting sequence), an application previously demonstrated [9]. The *attB*-flanked PCR products were generated in two sequential PCRs, with two sets of partially overlapping primers (see Methods section). The sequence of primers used for creating the current collection of functional modules is provided as Additional file 3: Table S1. Since all modules in each category (N- or C-terminal) were cloned the same way into pDONR-P4-P1R or pDONR-P2R-P3, they were fully exchangeable and conferred the desired combinatorial character to the toolkit.

**Table 1:** Plasmids encoding functional modules currently available on the toolkit

**5′ module**	**3′ module**
pDONR P4-P1R-**mKate2**	pDONR P2R-P3-**V5-6xHis**
pDONR P4-P1R-**V5-6xHis**	pDONR P2R-P3-**mKate2**
pDONR P4-P1R-**EGFP**	pDONR P2R-P3-**IRES_ECFP**
pDONR P4-P1R-**EYFP**	pDONR P2R-P3-**SV40 polyA signal**
pDONR P4-P1R-**ECFP**	pDONR P2R-P3-**EGFP**
	pDONR P2R-P3-**EYFP**
	pDONR P2R-P3-**ECFP**

Plasmids based on the pDONR P4-P1R vector contain inserts that will be placed at the 5′ flank of the cDNA and will encode the N-terminal module of the fusion protein. On the other hand, those based on pDONR P2R-P3 will transfer their insert to a position flanking the 3′-end of the cDNA, and will add a C-terminal module to the fusion protein in case they encode a peptide.

### Construction and expression of the fusion protein prototypes

**Figure 4:**
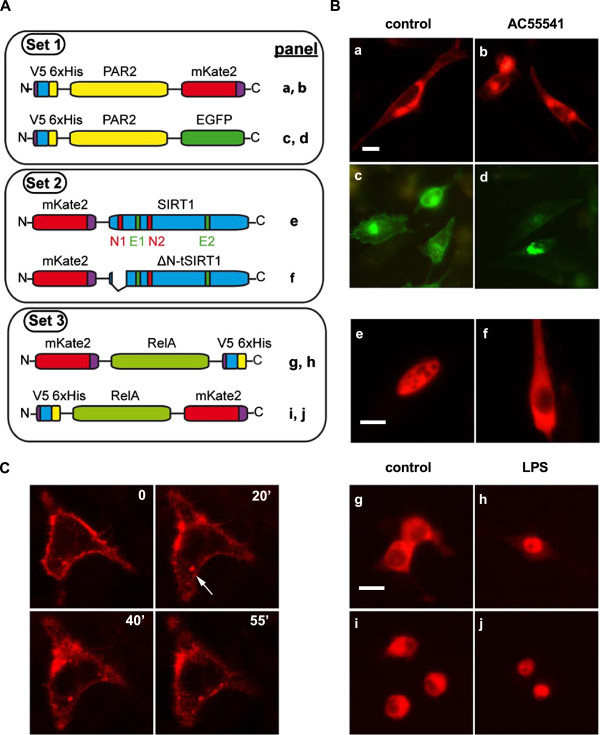
**Expression of fluorescent fusion proteins. A**. Schematic representation of the fusion proteins produced in order to test the feasibility of our toolkit, indicating the micrograph panels in Figure 4B where each protein’s expression is shown. The relative positions of the nuclear location signals (N1, N2) and nuclear export signals (E1, E2) in murine wild type (wt) SIRT1 is shown. *ΔN-tSIRT1,* N-terminal-deleted murine SIRT1 (see main text); the portion deleted from the wt isoform is indicated. Fusion proteins are clustered in functional sets, as described in the main text. **B**. Detection of fluorescent protein fusions under an epifluorescence microscope. Panels a-d: expression of PAR-2 fused to mKate2 **(a,b)** or EGFP **(c,d)** in transiently-transfected HeLa cells under control conditions **(a,c)** or after treatment with the PAR2-specific agonist AC55541 (5 μM, 1 h; panels **b**, **d**). White bar: 10 μm. Panels **e**, **f**: Differential localisation of wt- **(e)** or *ΔN-tSIRT1***(f)** proteins fused to mKate2 in transiently-transfected HeLa cells, under control conditions. White bar: 10 μm. Panels **(g-j)**: expression of two p65/RelA-based fusions with mKate2 after transient trasfection of their expression vectors into Raw264.7 monocytes, under control **(g,i)** or LPS-stimulated conditions (100 ng/ml, 1 h; panels **h**,**j**). White Bar: 10 μm. All micrographs were taken at 200X magnification. ***C*.** Frames from a time-lapse movie of a Raw264.7 monocyte transfected with the expression vector for the PAR2-mKate2 fusion protein and imaged on a confocal microscope (63X objective lens) after adding 5 μM AC55541 in order to monitor cellular trafficking of the receptor. The complete time-lapse experiment is provided as Additional file 5: Movie S1. Time elapsed after adding the agonist is indicated in each frame. The white arrow in the 20 min-panel points at some PAR2-mKate2-labelled intracellular vesicles that are seen trafficking in Additional file 5: Movie S1.

We constructed a panel of vectors for expressing fusion proteins in order to test the feasibility of our cloning toolkit. These vectors were obtained through MultiSite Gateway recombination reactions between plasmids encoding the N-terminal modules, the ORFs of interest, the C-terminal modules, and the pEF5/FRT/V5/DEST-R4-R3 destination expression vector described above. LR clonase-mediated recombination between compatible *att* sites on the participating plasmids produced a chimeric ORF that substituted the engineered Gateway cassette on plasmid pEF5/FRT/V5/DEST-R4-R3. The *attB1/attB2* sites flanking the *attR4/attR3* Gateway cassette were not targeted by the LR-recombinase [13]. Expression of the ORFs produced fusion proteins where the three component parts were separated by peptide linker arms resulting from the translation of the *attB1* and *attB2* sites flanking the protein of interest (see above). Since the upstream *attB1* and *attB4* recombination sites are located between the EF1α promoter and the translation initiation codon of the N-terminal module, and the *attB3* and downstream *attB2* sites lay beyond the stop codon in the C-terminal module, none of them would participate in the translated product (Figure 2, panel 4). Even though there seems to have been no systematic analysis of the possible functional interference of the residual *attB1/attB2* sites in the expression of fusion proteins [10], individual studies found those sites to be either neutral [16] or detrimental [17] for protein expression, hence the need to assess their convenience for the intended applications. The fusion proteins obtained with this approach are listed on Table 2 and are grouped in sets that illustrate some of the possibilities of the cloning toolkit. The first set highlights the combinatorial aspect of the collection of functional modules, since it is composed by two fusion proteins that share the N-terminal and central (i.e. the protein of interest) modules but that are fused to different fluorescent proteins at the C-terminus. This would be helpful for optimising fluorescent fusion proteins since different combinations can be produced up front and tested for the existence of steric hindrance, poor fusion stability and functional interference by oligomerization of the fluorescent module [18]. Furthermore, it would also provide some flexibility for designing coexpression experiments, because fusion proteins with different emission spectra would be available to suit each case (See panel A, Additional file 4). This set comprises two fusion proteins containing the Proteinase-activated receptor PAR2 (V5-6xHis: PAR-2: mKate2 and V5-6xHis: PAR-2: EGFP, Figure 4A). The second set represents the possibility of generating mutations in the ORF of interest while still in the entry clone. In this way, the construction of one mutational library would be sufficient for preparing a range of expression vector libraries to be screened on different model systems (mammalian cells, yeast, etc.) or with different reporters. This would avoid the need to go through independent mutagenesis reactions for creating libraries based on different expression vectors (See panel B, Additional file 4), and should be helpful for studying the effects of point mutations or deletions on the function of the protein of interest (e.g. intracellular trafficking). This set comprises the wild type (wt) form as well as an N-terminal deletion of the mouse sirtuin SIRT1 (*ΔN-t-SIRT1,* Figure 4A), both N-terminally fused to mKate2. This deletion affects the first nuclear location signal (N1) and may produce alterations on the intracellular distribution of the protein [19]. Finally, the third set represents the two possible arrangements of a fusion protein with functional modules flanking the ORF of interest (See panel C, Additional file 4). The ability to generate the two proteins up front would facilitate the identification of the order that causes the least interference on the protein’s function. Our example consists of two fusion proteins where the far-red fluorescent protein mKate2 [20] and a V5-6xHis epitope tag cassette were placed either N-terminal or C-terminal relative to the human p65/RelA subunit of NFkB (mKate2: p65: V5-6xHis and V5-6xHis: p65: mKate2, Figure 4A).

**Table 2:** Panel of expression vectors encoding fusion proteins

**Vector**	**5′-module**	**Central module**	**3′-module**
**Set 1**			
pEF5/FRT-DEST-R4-R3_V5-6xHis: PAR2:mKate2	V5-6xHis	PAR-2	mKate2
pEF5/FRT-DEST-R4-R3_V5-6xHis: PAR2: EGFP	V5-6xHis	PAR-2	EGFP
**Set 2**			
pEF5/FRT-DEST-R4-R3_mKate2: SIRT1	mKate2	SIRT1	*SV40 polyA*
pEF5/FRT-DEST-R4-R3_mKate2: ΔNtermSIRT1^a^	mKate2	ΔNtermSIRT1	*SV40 polyA*
**Set 3**			
pEF5/FRT-DEST-R4-R3_mkate2: RelA:V5-6xHis	mKate2	p65	V5-6xHis
pEF5/FRT-DEST-R4-R3_V5-6xHis:p65:mKate2	V5-6xHis	p65	mKate2

MultiSite Gateway recombination reactions were carried out between the adapted destination expression vector pEF5/FRT-DEST-R4-R3, and plasmids encoding 5′-module, the ORF of interest (central module) and the 3′-module as described. The resulting expression plasmids are listed on the table, with an indication of the functional modules used for making each fusion protein, and are organised in sets, according to the toolkit properties intended to illustrate (see main text). The central module encoding wt SIRT1 is an entry clone obtained from an ORFeome library, used in the MultiSite cloning reaction with no prior modifications. The SV40 polyA module (italics) does not encode for a peptide but contains a polyA signal that precedes the BGH polyA signal provided by the pEF5/FRT-DEST-R4-R3 vector.

^a^The “Δ” symbol stands for “deleted”.

The constructs described above were transiently transfected into cells in order to test the feasibility of our approach (Figure 4B, panels a-j). In HeLa cells, C-terminal fusions of PAR-2 with mKate2 (Figure 4B, panels a, b) or with EGFP (panels c, d) were expressed separately in transient transfection experiments. Cells under control conditions showed localisation of PAR-2 fluorescent fusions mostly at a perinuclear compartment [21], with a weaker signal located near the plasma membrane (a, c). Treatment of the cells with the PAR-2-specific agonist AC55541 caused a slight redistribution of the fluorescent signal from either fusion protein that concentrated on a smaller area around the nucleus (b, d). Also in HeLa cells, expression of wt SIRT1 fused to mKate2 showed nuclear localisation (Figure 4B, panel e), but the loss of an N-terminal fragment of mSIRT1 encompassing N1, caused the fusion protein to be localised in the cytoplasm (Figure 4B, panel f), in agreement with previous data [19]. In Raw264.7 monocytes, both the mKate2: p65:V5-6xHis and V5-6xHis:p65:mKate2 fusions (Figure 4A, set 3) demonstrated cytoplasmic localisation under control conditions (Figure 4B, panels g, i), as expected [22]. LPS treatment of the cells induced nuclear translocation of both fusion proteins (Figure 4B, panels h, j). The existence of slight differences in the intracellular distribution of fluorescence between different fusions of the same ORF (compare panels a and c, or g and i) suggest there might be effects related to the fusion arrangements that would deserve further investigation. If confirmed, they would illustrate the usefulness of our cloning strategy. As we pointed out in the background section, the study of the intracellular behaviour of a newly-described protein may help delineate its possible functions. Our cloning toolkit would be useful for the generation of fusions to be specifically used in such studies. This is exemplified by Additional file 5: Movie S1, where the fusion of the monomeric fluorescent protein mKate2 to the C-terminus of PAR-2 was expressed in a Raw264.7 monocyte by transient transfection of the pEF5/FRT-DEST-R4-R3_V5-6xHis: PAR2:mKate2 expression vector, and observed under a confocal microscope in a time-lapse experiment aimed at tracking the protein’s behaviour following treatment with the PAR-2 specific agonist AC55541. Figure 4C provides a series of still frames extracted from the time-lapse recording at the indicated times after adding the agonist. These show the agonist-induced clustering and internalization of the plasma membrane-associated PAR-2:mKate2 fusion, as well as the presence of intracellular vesicles containing the fluorescent protein (Figure 4C, arrow), as previously described [21]. Active perinuclear trafficking of the vesicles can be appreciated in the complete time-lapse movie (Additional file 5: Movie S1). Additional file 6 Movie S2 corresponds to a shorter time-lapse sequence of the same cell recorded under control conditions, just before adding the agonist, and shows expression of the fusion protein in continuous association with the plasma membrane, as well as in some intracellular vesicles that display a more modest trafficking behaviour.

All the fusion proteins described above were expressed from pEF5/FRT/V5/DEST-R4-R3, a plasmid derived from pEF5/FRT/V5/DEST (see above and Methods section). This is a vector compatible with the FLP-In system (Life Technologies), which allows the generation of isogenic clones with stable plasmid integration by using a selection of cell lines that contain a single FRT recombination sequence in their genome (FLP-In cell lines). Thus, our system should facilitate the assembly of expression vector libraries consisting of fusions between selected functional modules and a library of randomized mutations of a cDNA, so they could be stably-transfected into an FLP-In cell line and screened for a mutation conferring the properties sought after, in the absence of clonal variegation caused by genome-positional effects. Furthermore, this would not be restricted to randomized mutation libraries, but could also be applied to libraries consisting of collections of cDNAs (e.g. the Gateway-based Human Kinase Open Reading Frame Collection from the Centre for Cancer Systems Biology (DFCI)/Broad Institute (MIT) [23]). Our system would also be useful for the generation of chimeric proteins. Thus, a protein coding sequence could be divided into three fragments at appropriate sites that did not compromise the main structure or function of the protein, with each fragment cloned into one of the three pDONR vectors in the toolkit. Different isoforms in a family of proteins could be cloned in this way so that homologous domains cloned in the same type of pDONR vector could be shuffled between the isoforms in order to analyse the impact on the proteins’ function. Similarly, a collection of mutants from a single domain could be cloned with the remainder of the wild type ORF in order to screen for residues involved in a specific function. Although we have not addressed these specific applications, pioneering work on the yeast STE2 receptor by Cheo and colleagues with an analogous set up [9] suggests that our design could be used in such studies.

Interestingly, some of the clones encoding functional modules in our toolkit could be exchanged with those from similar platforms built with the same MultiSite Gateway cloning kit as ours, expanding the number of possible fusion protein combinations. One example is the platform known as Tol2Kit [24]. It consists of a series of 5′-, middle- and 3′-clones arranged in the context of several destination vectors specific for zebra fish transgenesis, and was created to facilitate the obtention of strains expressing a variety of minigene constructs consisting of *promoter_coding sequence_3′tag* arrangements [24]. Another example is the pTransgenesis system [25], which consists of collections of entry vectors initially developed to facilitate the construction of minigenes to be expressed in transgenic *Xenopus* strains, but are also compatible with transgenesis in other model organisms such as *Drosophila*, zebrafish and mammalian cell models, thus facilitating the study of the evolutionarily conserved properties of biological systems [25]. Finally, Nagels-Durand and colleagues have recently reported a novel set of destination vectors specific for expression in *Saccharomyces cerevisae* that allow MultiSite recombination cloning of promoters, ORFs and epitope tags in combination with a choice of auxotrophy markers and replication mechanisms in this model organism [26]. Some of the functional modules described in our work overlap with those from these other platforms (Table 1 and [24-26]), and have been successfully used for the applications described therein. Fluorescent protein-encoding modules from our toolkit as well as from the other platforms mentioned above could also be used as reporters in new projects aimed at characterizing ORFeome-inspired promoteromes, such as in the pioneering work of Denis Dupuy and collaborators [27], who constructed a library of intergenic regions comprising proximal transcriptional regulatory elements plus the 5′UTRs cloned in the pDONR P4-P1R vector, and used it in combination with ORFs derived from the nematode’s ORFeome in Multisite Gateway cloning reactions that generated vectors with a *promoter:ORF:reporter* arrangement. These were used subsequently in studies of the regulatory mechanisms controlling gene transcription and protein localization at genome-level [27,28].

Despite the availability of a more recent version of the MultiSite Gateway cloning kit (*MultiSite Gateway Pro*) that allows using any single-fragment destination vector in two-, three-, or four-fragment recombination reactions without further modification [29], we chose the earlier version of the kit because this would allow the use of entry vectors containing the *attL1/attL2*-flanked ORF of interest as obtained from public sources, without further subcloning. This is precluded in the Pro version of the kit, since those *att* sites ought to lie on separate entry vectors because of the kit’s configuration. Thus, with a *MultiSite Gateway Pro*-based cloning platform, any *attL1/attL2* flanked-ORF of interest obtained from a public repository would still have to be PCR-amplified and subcloned into at least one pDONR vector appropriate for the Pro version of the kit prior to the MultiSite recombination reaction, blunting the advantage conferred by the ready availability of the entry clone. In our approach, engineering is instead restricted to the destination vector of choice, which once adapted for MultiSite Gateway recombination can be used to generate multiple fusion proteins with the unmodified entry clones from the library. Nevertheless, cloning platforms that use the MultiSite Gateway Pro kit are perfectly possible and one such has been developed [30], which allows the versatile creation of minigenes to be used in *Drosophila* transgenesis for the tissue-specific expression of proteins, including fluorescent reporters. Platforms reported in earlier publications, such as the *Drosophila* Gateway vector collection devised by the Murphy lab at the Carnegie Institution [31], and the *Saccharomyces cerevisae* collection of Gateway destination vectors constructed at the Lindquist lab [32] also allow directly producing fusion proteins with both custom made-, or ORFeome-derived entry clones, since their input consists of plasmids containing ORFs flanked by *attL1/attL2* sites, which are used in single-fragment LR recombinations to generate the expression vectors. In these platforms, though, variability lies with the destination vectors, which contain functional modules preinserted at 5′ or 3′ relative to the standard Gateway cassette, which will be expressed in-frame with the Gateway-shuttled ORF of interest. Since the functional modules lie beyond the *att* sites, construction of these platforms required complex cloning of Gateway cassette-derivatives into a large number of expression vectors in order to achieve the desired number of module combinations.

Although toolkits for fusion protein construction based on sequence-directed cloning methods provide versatility and ease of use, the possible functional impact of peptide “scars” linking the modules (resulting from the translation of intervening residual cloning sites, see above), needs to be taken into account [33]. In view of this, other more economical, non-sequence-directed, “seamless” recombinational cloning methods for joining two or more DNA fragments without the operation of extraneous sequences would represent an attractive alternative [34,35]. Nevertheless, in the most widely used seamless methods, DNA fragments to be assembled ought to be amplified by PCR with primers that provide some sequence overlap at their ends. The distribution of these sequence overlaps among the fragments ensures they are stitched together in the required order. In other instances, “stitching” oligonucleotides can be used that act as a bridge encompassing the ends of the unrelated sequences to be joined [35]. In either case, the generation of a combinatorial cloning platform based on such methods would involve prior knowledge of the fusions that would be of interest in order to provide necessary tools in advance. This would somewhat curtail the implementation of high-throughput strategies based on the use of ORFeome-derived clones, as it would require a case-based design, which is not necessary when universal external connectors (e.g. *att* sites) are used. Furthermore, PCR-amplification of modules for constructing specific fusions would require checking every resulting chimeric cDNAs for the presence of unwanted mutations, while such controls are only required at the time of subcloning if the same modules are perpetuated as inserts of a plasmid in the context of a Gateway-based platform. In the absence of a perfect recombination-based solution, one ought to consider what is best for a given application, whether lowering the risk of encountering scar-derived functional effects, or using high throughput applications. Nevertheless, this is no obstacle to using both approaches in a complementary way since, for instance, the assembly of composite modules derived from the sequence of two or more proteins could be achieved by using seamless recombinational cloning, and the new modules could then be incorporated into Gateway-based cloning platforms for their fusion to other peptides. Interestingly, non-recombination-based cloning platforms such as Golden Gate, Golden Braid, and MoClo [33,36,37], which have shown great potential for scalability in the construction of multipartite expression vectors, could also be adapted for the seamless cloning of fusion proteins with the possibility of using peptide-encoding modules in high-throughput applications. Careful design of the module-cloning strategy may allow the construction of fusion proteins with a minimal contribution of scar sequence between the modules [33,36]. The downsides are that these systems require that cloned DNA fragments are free from the infrequent target sites for the type IIS restriction enzymes they are based upon (this may require the fragment’s sequence to be modified prior to cloning), and that the ORFs of interest cannot be used straight from Gateway-based ORF collections but would require PCR-amplification with primers providing the appropriate restriction enzyme sites. All in all, the above considerations should be a guide for the future development of cloning strategies with the potential to use peptide-encoding modules across different platforms.

## Conclusions

We describe the blueprint for a system that streamlines the cloning of fusion proteins from Gateway-based, ORF-containing entry clones. It is based on sets of plasmids that encode customized functional modules to be fused to an ORF of interest, plus an adaptor plasmid that allows existing destination expression vectors to participate in MultiSite cloning reactions leading to the cloning of fusion proteins. Multiple fusions can thus be potentially assembled from a single ORF in a combinatorial way, and expressed in diverse cellular models. Expression vectors are easy to construct and update, since cloning is based on Gateway technology. We believe this approach will be widely useful for the scientific community as it should extend the range of possible studies based on fusion proteins beyond those currently afforded by cloning vectors with preinserted modules. Plasmids will be made available through Addgene.

## Methods

### Molecular biology

PCR reactions were carried out with AccuPrime Pfx SuperMix (Life Technologies) as indicated by the manufacturer. The pDONR plasmids and cloning vector pDEST-R4-R3 were obtained as part of the MultiSite Gateway cloning kit (Life Technologies catalog # 12537023). Destination expression vector pEF5/FRT/V5-DEST was obtained from Life Technologies (catalog # V6020-20). This is a vector for cloning and expressing proteins in FLP-In™ isogenic cell lines. The expression of cloned proteins is driven by the human elongation factor 1α promoter located upstream of the Gateway cassette. The vector has an FRT recombination site that mediates FLP recombinase-directed integration of the vector into a unique homologous FRT site in the genome of FLP-In™ cell lines, and the hygromycin resistance gene acts as a selectable marker for integration. Nevertheless, it behaves just as any other expression vector in transient transfection experiments. BP- and LR-recombination reactions were carried out with BP clonase II and LR Clonase II Plus enzyme mixes, respectively (Life Technologies), following the manufacturer’s instructions. Reactions were stopped by addition of Proteinase K and incubation at 37°C for 10 min. All competent *E. coli* strains were obtained from Life Technologies and used in one-shot format for plasmid transformation. LB medium was supplemented where indicated with selection antibiotics at the following concentrations: ampicillin (100 μg/mL), kanamycin (50 μg/mL), chloramphenicol (50 μg/mL).

### Site-directed mutagenesis of the chloramphenicol-acetyl transferase (*CAT*) gene in the Gateway cassettes

Inactivation of the type I *CAT* gene in the Gateway selection cassette of pDONR221 and pEF5/FRT/V5-DEST was achieved by site-directed mutagenesis. The oligonucleotides 5′-CCCCCGTTTTCACCTAAGGCAAATATTATAC-3′ (forward) and 5′-GTATAATATTTGCCTTAGGTGAAAACGGGGG-3′ (reverse) were used to introduce a nonsense mutation, replacing methionine 173 with a stop codon (underlined). This was designed to cause the premature interruption of the 219-residue type I CAT protein, resulting in the loss of the c-terminal α-Helix 5 [38]. This region allows the formation of the trimeric complex of identical subunits that constitutes the functional enzyme, having additional positive effects on the protein’s solubility [39]. The mutation was expected to disrupt these properties and have profound deleterious effects on the enzyme’s activity. The mutagenesis oligonucleotides were designed so that an *NcoI* site would be destroyed as a consequence of the mutation, which could then be used for diagnostic purposes. The oligonucleotides were used in combination with the QuikChange Lightning site-directed mutagenesis kit (Agilent), with the exception that transformation of the mutagenesis reactions was done into ccdB Survival™ 2 T1R competent cells to avoid the lethality of the *ccdB* gene in the Gateway selection cassette. After transformation, the cultures were spread on LB/agar dishes containing either ampicillin (pEF5/FRT/V5-DEST) or kanamycin (pDONR221) to allow the growth of clones containing the putatively mutant plasmids, since resistance to these antibiotics would be unaffected. 10–20 Colonies were picked and sequentially streaked first onto LB/agar Petri dishes supplemented with ampicillin or kanamycin, and secondly onto dishes additionally supplemented with chloramphenicol, in order to identify colonies that were sensitive to this antibiotic as a result of the mutation in the *CAT* gene. Colonies that failed to grow in the ampicillin/chloramphenicol (pEF5/FRT/V5-DEST) or kanamycin/chloramphenicol (pDONR221) dishes were identified, and their counterpart streak on the dish with a single antibiotic was used to rescue the clone and prepare plasmid. Since chloramphenicol sensitivity (Cam^S^) could also result from spontaneous mutation of the *CAT* gene, the presence of the desired mutation was confirmed by digestion of the plasmids with *NcoI* and by sequencing. The resulting chloramphenicol-sensitive, mutant plasmids pDONR221_Cam^S^ and pEF5/FRT/V5-DEST_Cam^S^ were subsequently used in recombination reactions for the incorporation of the *attR4-attR3* Gateway cassette.

### Construction of pDONR221-R4-R3

The standard Gateway cassette in pDONR221 was replaced by the *attR4-attR3* MultiSite Gateway cassette, resulting in vector pDONR221-R4-R3. This was done by using the methodology described by Magnani et al. [13], with modifications. Firstly, we PCR-amplified the *attR4*-*attR3*-flanked MultiSite Gateway cassette from vector pDEST-R4-R3 [13] in two sequential PCR reactions, by using oligonucleotide primers that added external *attB1* and *attB2* sites in juxtaposition to the existing *attR4* and *attR3* sites, respectively. The first PCR (10 cycles) was performed with the oligonucleotide primer pair: 5′-AAAGCAGGCTCAACTTTGTATAGAAAAGTTG-3 (*attR4fw*) and

5′-AAAGCTGGGTCAACTATGTATAATAAAGTTG-3′ (*attR3rv*), while the second PCR reaction (32 cycles) was carried out with the external primer pair: 5′-GGGGACAAGTTTGTACAAAAAAGCAGGCTCA-3′ (*attB1-R4fw*) and 5′-GGGGACCACTTTGTACAAGAAAGCTGGGTCA-3′ (*attB2-R3rv*), by using a 1:10 dilution of the first PCR reaction in fresh Accuprime *Pfx* supermix as the source of template. The final PCR product, which contained an intact *CAT* gene, was used with plasmid pDONR221-Cam^S^ in a BP recombination reaction for 1h at 25 °C. After transformation of the reaction into ccdB Survival™ 2 T1R competent cells, the culture was plated onto an LB/agar dish containing kanamycin and chloramphenicol to select for colonies harbouring plasmids that had successfully recombined with the PCR product and were able to grow in the presence of chloramphenicol. Colonies were picked for preparation of plasmid and further analysis. The presence of the *attR4* and *attR3* sites was confirmed by sequencing of the vector. The integrity of the *ccdB* gene in pDONR221-R4-R3 was tested by transformation of the vector into Top10 competent cells, which are sensitive to the *ccdB* gene and failed to grow in the presence of the vector.

### Construction of pEF5/FRT/V5-DEST-R4-R3

Adaptation of pEF5/FRT/V5-DEST for MultiSite Gateway cloning was carried out as described by Magnani et al. [13], with modifications. Briefly, an LR recombination reaction was carried out at 25°C for 16h between plasmids pEF5/FRT/V5-DEST_Cam^S^ (see above) and pDONR221-R4-R3. Importantly, the *attR4/attR3* sites in pDONR221-R4-R3 cannot recombine with the juxtaposed *attL1/attL2* sites [13]. After transformation of the reaction into ccdB Survival™ 2 T1R competent cells, the cultures were plated onto LB/agar dishes containing ampicillin and chloramphenicol to select for colonies harbouring the recombined pEF5/FRT/V5-DEST plasmid. In this way, the *attR1/attR2*-flanked standard Gateway cassette in pEF5/FRT/V5-DEST was substituted by the *attR4/attR3*-flanked MultiSite Gateway cassette, obtaining pEF5/FRT/V5-DEST-R4-R3, which was ready for a MultiSite LR recombination cloning reaction with the three modules that would make up the fusion protein of choice.

### Entry clones containing the ORFs of interest

The entry clones for p65 and PAR-2 were generated by BP clonase-mediated recombination reactions (1 h, 25°C) between vector pDONR221 and purified PCR-products representing each of the full-length cDNAs. Nevertheless, purification of PCR products prior to BP cloning is not essential as the reactions also work well with non-purified PCR products. To make those entry clones apt for optional single-fragment recombination into a standard destination vector, part of a Kozak sequence (GCCGCC) was included in the forward primers, 5′to the ATG initiation codon, in order to optimise translation of the cDNA. Thus, when the same entry clone participated as a central element in the cloning of a fusion protein, the Kozak sequence translated into two alanine residues preceding the initial methionine. The stop codon was omitted from the cDNA-specific sequence of the reverse primers so as to allow the construction of fusions with a C-terminal module. The PCR products were generated by using a two-step PCR method similar to that used to amplify the *attR4*-*attR3*-flanked MultiSite Gateway cassette. In the first PCR reaction, a set of primers that contained part of the *attB1* (forward primer) or *attB2* (reverse primer) recombination sites followed by template-specific sequence (Additional file 3: Table S1), was used in combination with a template-containing plasmid, in a 10-cycle PCR reaction. A second, 32-cycle PCR reaction was set up by diluting 1/10 the first PCR reaction, and supplementing it with a new set of primers (Additional file 3: Table S1) that were complementary to the partial *attB1* (forward primer) or *attB2* (reverse primer) sequences incorporated into the PCR product by the first set of primers, and that extended the recombination sites to the full length recommended by Life Technologies for BP clonase-mediated cloning. This two-step method allowed the use of the second set of primers in multiple cloning projects since the specificity with regard to the cDNA relied on the sequence of the first set of primers, resulting in potential savings in the costs of oligonucleotide synthesis in the long term. It should be noted that this is not a technical requirement since one-step PCR reactions with primers providing full-length, flanking *attB* sites are widely used for Gateway cloning of PCR products. The cDNA fragment comprising positions 141 to 1793 of the human p65/RelA mRNA sequence (accession number NM_021975.3), excluding the natural stop codon, was amplified as described above from plasmid pCDNA3.1-p65 [40], with the primers indicated in Additional file 3: Table S1. The mouse thrombin receptor PAR-2 cDNA was amplified from the FANTOM Full Length cDNA clone number G8300117P07 (pFLCI-PAR2, The Institute of Physical and Chemical Research (RIKEN) [41]), with primers (Additional file 3: Table S1) that encompassed the sequence between positions from 114 to 1313 in the mouse mRNA (accession number NM_007974). For the fusion protein that contained the full-length mouse SIRT1 coding sequence, we used an IMAGE ORFeome collaboration clone (100066295) with the SIRT1 cDNA already flanked by *attL1/attL2* sites in vector pENTR223-SfiI [42]. Cultures of bacteria transformed with this plasmid were grown with LB medium containing 50 μg/ml spectinomycin. A mouse SIRT1 cDNA with a deletion that affected a region near the N-terminus of the protein (*ΔN-t-SIRT1)* was amplified from plasmid pUSEamp SIRα2 (Millipore). This internal deletion affected residues Leu7 to Ala123 but did not disrupt the rest of the ORF, causing the loss of a region encompassing the N-terminal nuclear location signal NLS1 [19]. The deletion was caused by off target annealing of the forward primer in the first PCR reaction (Additional file 3: Table S1), to a sequence lying 354 bp downstream of the target sequence. The full *attB1* and *attB2* sites were completed in a second PCR by using the universal set of primers, and the PCR product was cloned into pDONR221 through BP clonase-mediated recombination, as described above. All PCR primers used were designed so that the eventual *attB1/attB2*-flanked PCR products would be in frame in the context of three-fragment recombinations for the production of fusion proteins. The sequence of all clones was verified by sequencing.

### Construction of the modules’ collections

The collections of N-terminal and C-terminal modules were constructed as clones containing DNA inserts in vectors pDONR P4-P1R and pDONR P2R- P3, respectively. The inserts (italics) were PCR-amplified from the following plasmid templates: *mKate2*, pmKate2-C (Evrogen); *EGFP*, pLV-EGFP (kind gift from M. Perez-Pinzon, UM); *EYFP*, pEYFP-mito (Clontech); *ECFP* and *IRES_ECFP*, pYIC (Addgene plasmid 18673 [43]; *V5-6xHis epitope tag cassette*, pEF6/V5-His (Life Technologies). Construction of the clone containing the SV40 early polyadenylation signal has previously been described [44]. All inserts were amplified in a two-step PCR reaction, similarly to the way described in the section above, with the primers described in Additional file 3: Table S1. In this case, the primers attached flanking *attB4/attB1R* sites to the PCR products to be cloned into pDONRP4-P1R, while those to be cloned into pDONRP2R-P3 were furnished with *attB2R/attB3* sites. When the functional modules were cloned into pDONR-P4-P1R (N-terminal modules), the forward primer in the first pair contained a Kozak sequence in order to improve translation of the fusion protein. Furthermore, if the template sequence contained a stop codon, this was not included when designing the reverse primer of the first pair so as to avoid interrupting translation downstream of the module. Nevertheless, when the module was to be located at the C-terminal end of the fusion (a pDONRP2R-P3-based clone), the stop codon was allowed into the sequence of the reverse primer of the first pair. Cloning of the PCR products into their corresponding pDONR vector was carried out by using BP clonase, as described above. The sequence of all clones was verified by sequencing.

### Cloning of fusion proteins into pEF5FRTV5DEST-R4-R3

Chimeric ORFs for the expression of fusion proteins were assembled by performing MultiSite LR recombination reactions between the three selected entry clones and the adapted pEF5FRTV5DEST-R4-R3 destination expression vector. Ten femtomoles of Maxiprep-quality DNA from each entry vector were mixed with 20 femtomoles of the destination expression vector and 2 μl of LR Clonase II plus enzyme mix (Life technologies) in a final volume of 10 μl and incubated for 16 h at 25°C, following instructions from the manufacturer. A 2-μl aliquot of the reaction was transformed into either Top10 or stbl3 *E. coli*, and the reaction was plated on LB-agar medium supplemented with ampicillin to select transformed bacteria. Colonies potentially containing recombined pEF5FRTV5DEST expression vectors with the chimeric ORF were picked with sterile pipette tips and streaked onto Petri dishes containing LB-agar medium plus ampicillin in order to amplify them. These cultures were subsequently streaked on Petri dishes containing LB-agar medium supplemented with ampicillin and chloramphenicol in order to check for the presence of colonies containing non-recombined destination expression vector that may have spontaneously mutated the *ccdB* gene in the Gateway cassette. The same colonies were also tested for growth on plates with LB-agar medium containing the antibiotic to which the entry clones that had been used in the LR reaction conferred resistance to (kanamycin, spectinomycin), since we observed an occasional phenomenon of cotransformation of the destination expression vector with the entry clones. This was observed even though both the pDONR series of plasmids and pEF5FRTV5DEST contain the same origin of replication and thus belong to the same incompatibility group. This is a phenomenon that has been thoroughly described elsewhere [45,46], and needs to be taken into account as it could be a confounding factor in plasmid preparations that are destined to be transfected. Future refinements of this method will have to be devised in order to avoid such cotransformation events in high-throughput applications. Only the colonies that grew exclusively on LB plus ampicillin were used for further tests. In our hands, screening of about 20–25 colonies per transformation was sufficient to find colonies harbouring the expression vector in the absence of “piggybacking” entry clones. The integrity of the chimeric ORFs in the recombined expression vectors was checked both by restriction digest and sequencing. As a guide to the expected colony yield, colony counting after transformation of half the reaction volume in a dedicated series of reactions resulted in 258 ± 54 (mean ± SD, n = 5) colonies per Petri dish. This number is lower than the range suggested by Life technologies in the Multisite Gateway cloning kit user’s manual (1000–5000 colonies, when the whole transformation is plated), which could be caused by procedural differences introduced as a result of our toolkit’s design. In any case, colony numbers expected from Multisite Gateway reactions are still below those produced in single-fragment LR recombinations (see Life Technologies’ Gateway user manuals) because of the participation of more DNA fragments, which should be taken into account when planning Multisite Gateway LR recombination reactions.

### Expression of fusion proteins

Expression vectors encoding chimeric ORFs were introduced into HeLa cells or Raw264.7 murine monocytes, in transient transfection experiments. Both cell lines were kindly provided by Dr Andy Clark (University of Birmingham, UK). In the case of HeLa cells, 1.5×10^4^ cells per well were seeded on top of glass coverslips in 24-well plates. Cells were transfected with expression vectors for mKate2 fused to wt SIRT1 or ΔN-t-SIRT1 (both with N-terminal mKate2), or PAR-2 (with mKate2 fused to the C-terminus of PAR2), as well as for PAR-2 fused to EGFP (also in a C-terminal fusion to PAR-2). One μg of plasmid DNA was transfected with 3 μl of Lipofectamine reagent (Life Technologies), following the manufacturer’s instructions. Forty-eight hours after transfection, cells transfected with the PAR-2 fluorescent fusions were either left untreated (control) or treated for 1 h with the PAR-2 specific agonist AC55541 (Tocris) at a 5 μM final concentration. At the end of the treatments, cells were fixed with 4% paraformaldehyde, rinsed in cold methanol followed by a brief wash in H_2_O and mounted in Fluoromount G (Southern Biotech). Cells were observed under a Nikon Eclipse E1000 fluorescence microscope. For the Raw264.7 cell line, 10^5^ cells per well were seeded on 24-well plates. Cells were transfected with 1 μg of maxiprep-quality plasmid DNA and 3.2 μl of jetPEI-Macrophage reagent (Polyplus transfection) per well, according to the manufacturer’s instructions. The transfected expression vectors encoded for fusions of p65 with the red fluorescent protein mKate2. Forty-eight hours after transfection, cells were either left in control conditions or treated with LPS, and were observed live under an Olympus IX70 fluorescence inverted- microscope. For the time-lapse experiment, Raw 264.7 cells seeded on a 25 mm Ø glass coverslip in a well of a six-well plate were transfected with the expression vector for the PAR2: mKate2 fusion protein and mounted in a chamber equipped with an incubation system with temperature control, in a Leica TCS-SL confocal inverted microscope (63X objective lens). A transfected cell was imaged live, with micrographs taken at 20 sec intervals, both under control conditions or after treating with 5 μM AC55541.

## Competing interests

The authors declare that they have no competing interests.

## Authors’ contributions

TS and AMP conceived the idea. TS designed the toolkit. RB, NI and TS carried out the cloning and transfection experiments and analyzed the data. TS and AMP wrote the paper. All authors read and approved the final manuscript.

## Supplementary Material

Additional file 1: Figure S1 Single fragment and MultiSite Gateway recombinational cloning. In the Gateway system*,* DNA fragments (such as a cDNA) can be PCR-amplified with primers that attach flanking *attB1/attB2* sites (B1, B2), and cloned into a compatible vector by carrying out a BP recombination (BP rec.). This generates a so-called Entry clone where the DNA fragment is flanked by *attL1/attL2* sites (L1, L2), and that can be subsequently used to shuttle the DNA fragment into destination vectors that provide specific functions. In standard single-fragment Gateway cloning, an *attL1/attL2*-flanked cDNA in the example is transferred to a destination vector that contains compatible *attR1/attR2* sites through an LR-recombination reaction (LR rec.). On the other hand, in the MultiSite Gateway cloning system, three different entry clones with DNA fragments flanked by sequence variants of the *attL* and *attR* sites (L3, L4, R3, R4) participate in a multi-fragment LR-recombination reaction with the promoter-less destination vector pDEST-R4-R3. This vector contains a Gateway cassette that is flanked by *attR4/attR3* sites, which conditions the order of recombination of the three fragments in the resulting destination vector owing to the nature of their respective flanking *att* sites, as indicated. Click here for file

Additional file 2: Figure S2 Mutation of the *CAT* gene in the Gateway cassette of pEF5FRT-DEST abolishes resistance to chloramphenicol. Cultures of ccdB-resistant *E. coli* transformed with pEF5FRT-DEST encoding a wild type (a,c), or a mutant version of the *CAT* gene (b, d), were streaked on LB-agar dishes containing ampicillin (a,b) or ampicillin plus chloramphenicol (c,d). While bacteria transformed with either of the plasmids were able to grow in the presence of ampicillin, further supplementation of the medium with chloramphenicol specifically prevented the growth of bacteria transformed with the plasmid containing the mutation of the *CAT* gene (d). Click here for file

Additional file 3: Table S1 Sequence of the oligonucleotides used for PCR amplification. Two-step PCRs were set up with the primers indicated on the table (fw: forward, rv: reverse) in order to attach the appropriate *attB* sites to the functional modules to be cloned by BP clonase-mediated recombination. The module-specific primers were used in the first PCR and contain part of the *att* sequence. The universal external primers were used in the second PCR to complete the *att* sites. In the module-specific primers, sequence in capitals corresponds to the oligonucleotide segment that anneals to the template, while the sequence in bold type is annealed by the universal external primer that will complete the corresponding *att* site. The same forward and reverse primers were used for the PCR amplification of EGFP, ECFP and EYFP, since the mutations dictating the fluorescence wavelength lie beyond the sequence annealed by the primers. The N-terminal V5-6xHis module was PCR-amplified with a three-step PCR. The first forward module-specific primer (a) attached a Kozak sequence and an initiation methionine codon to the cassette containing the epitope tags, but no *att*-related sequence (∅), while the second PCR was carried out with a second forward primer (b) that provided the seed for the *attB4* site. This site was completed in the last PCR, which was carried out with the corresponding external universal primers. Only one reverse module-specific primer was used in the first and second PCRs for this module. Click here for file

Additional file 4: Figure S3 Versatility of the cloning toolkit. *A*. Simultaneous construction of vectors expressing versions of the same fusion protein coupled to different fluorescent modules, offering a choice of optical properties in experiments where individual or multiple fusion proteins are expressed. *B*. A library of mutations can be generated in the vector encoding the ORF of interest so recombination of the library with intact functional modules would allow the generation of a homogeneous range of expression vector mutation libraries to be screened on different model systems. *C*. Fusion proteins can be constructed so that the functional modules flanking the ORF of interest are in either of the two possible orders, to evaluate putative effects on protein function. Click here for file

Additional file 5: Movie 1 Complete time-lapse sequence recorded on the PAR2:mKate2-expressing cell shown in Figure 4C. The sequence spans an almost one-hour period, with the AC55541 agonist added right at the start. Click here for file

Additional file 6: Movie 2 Time-lapse recording of the same cell as in Additional file 5: Movie 1, recorded under control conditions for 11 min before adding the AC55541 agonist. Click here for file
