# Supplementary figures and images for: A plasmid toolkit for cloning chimeric cDNAs encoding customized fusion proteins into any Gateway destination expression vector
Source: BMC Mol Biol. 2013 Aug 20;14:18. doi: 10.1186/1471-2199-14-18 (PMC3765358; doi:10.1186/1471-2199-14-18)

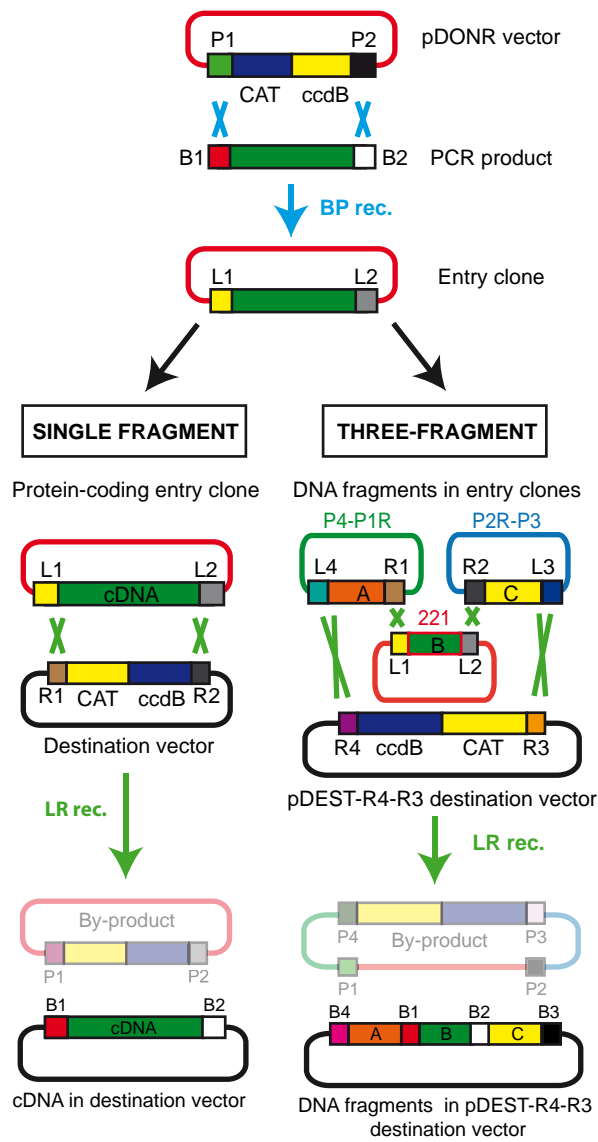

Supplement: Additional file 1: Figure S1 — Single fragment and MultiSite Gateway recombinational cloning. In the Gateway system, DNA fragments (such as a cDNA) can be PCR-amplified with primers that attach flanking attB1/attB2 sites (B1, B2), and cloned into a compatible vector by carrying out a BP recombination (BP rec.). This generates a so-called Entry clone where the DNA fragment is flanked by attL1/attL2 sites (L1, L2), and that can be subsequently used to shuttle the DNA fragment into destination vectors that provide specific functions. In standard single-fragment Gateway cloning, an attL1/attL2-flanked cDNA in the example is transferred to a destination vector that contains compatible attR1/attR2 sites through an LR-recombination reaction (LR rec.). On the other hand, in the MultiSite Gateway cloning system, three different entry clones with DNA fragments flanked by sequence variants of the attL and attR sites (L3, L4, R3, R4) participate in a multi-fragment LR-recombination reaction with the promoter-less destination vector pDEST-R4-R3. This vector contains a Gateway cassette that is flanked by attR4/attR3 sites, which conditions the order of recombination of the three fragments in the resulting destination vector owing to the nature of their respective flanking att sites, as indicated. [file 1471-2199-14-18-S1.pdf]

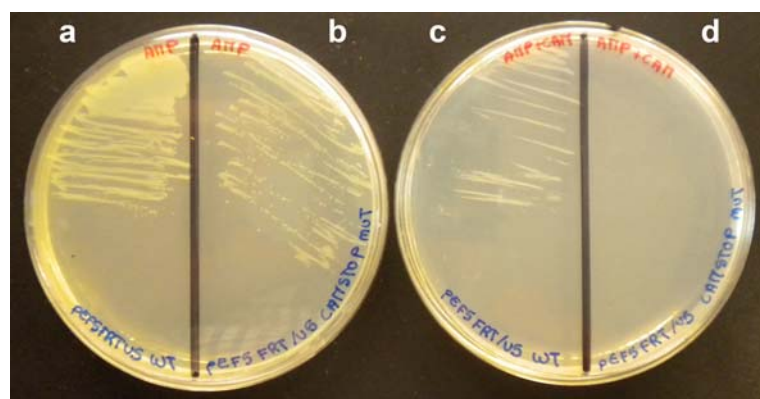

Supplement: Additional file 2: Figure S2 — Mutation of the CAT gene in the Gateway cassette of pEF5FRT-DEST abolishes resistance to chloramphenicol. Cultures of ccdB-resistant E. coli transformed with pEF5FRT-DEST encoding a wild type (a,c), or a mutant version of the CAT gene (b, d), were streaked on LB-agar dishes containing ampicillin (a,b) or ampicillin plus chloramphenicol (c,d). While bacteria transformed with either of the plasmids were able to grow in the presence of ampicillin, further supplementation of the medium with chloramphenicol specifically prevented the growth of bacteria transformed with the plasmid containing the mutation of the CAT gene (d). [file 1471-2199-14-18-S2.pdf]

**A**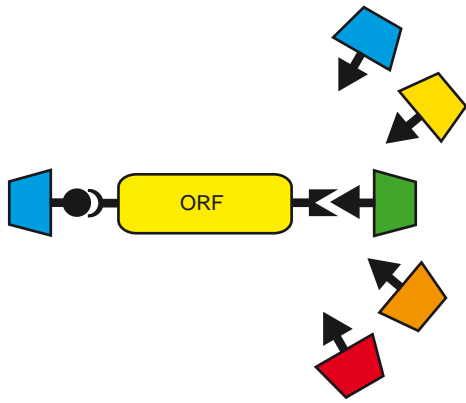**B**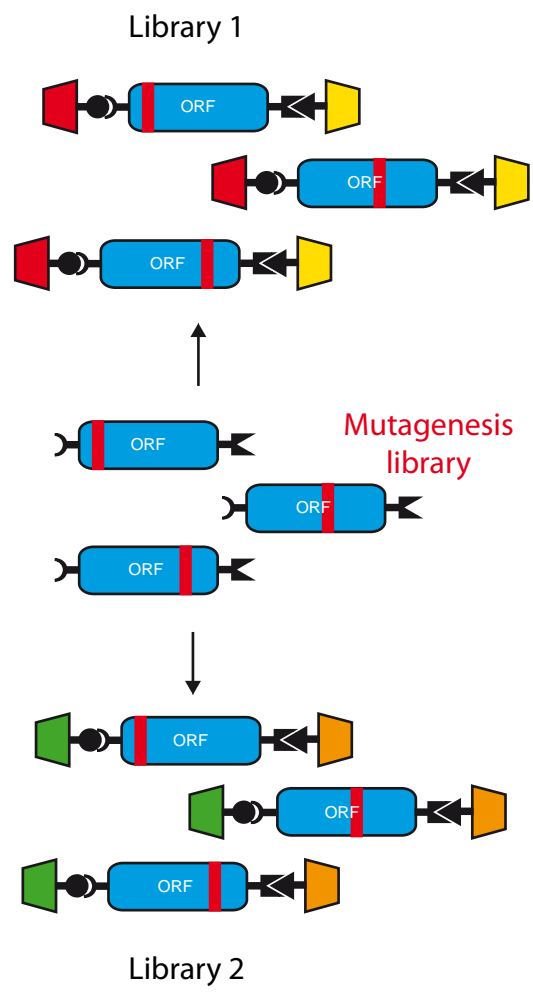**C**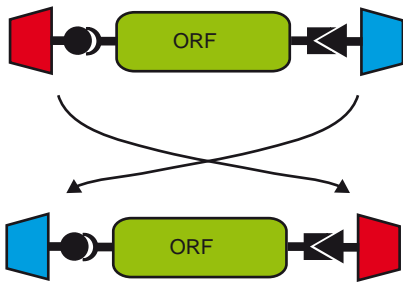

Supplement: Additional file 4: Figure S3 — Versatility of the cloning toolkit. A. Simultaneous construction of vectors expressing versions of the same fusion protein coupled to different fluorescent modules, offering a choice of optical properties in experiments where individual or multiple fusion proteins are expressed. B. A library of mutations can be generated in the vector encoding the ORF of interest so recombination of the library with intact functional modules would allow the generation of a homogeneous range of expression vector mutation libraries to be screened on different model systems. C. Fusion proteins can be constructed so that the functional modules flanking the ORF of interest are in either of the two possible orders, to evaluate putative effects on protein function. [file 1471-2199-14-18-S4.pdf]
