# Supplementary material for: A plasmid toolkit for cloning chimeric cDNAs encoding customized fusion proteins into any Gateway destination expression vector
Source: BMC Mol Biol. 2013 Aug 20;14:18. doi: 10.1186/1471-2199-14-18 (PMC3765358; doi:10.1186/1471-2199-14-18)
Supplement: Additional file 3: Table S1 — Sequence of the oligonucleotides used for PCR amplification. Two-step PCRs were set up with the primers indicated on the table (fw: forward, rv: reverse) in order to attach the appropriate attB sites to the functional modules to be cloned by BP clonase-mediated recombination. The module-specific primers were used in the first PCR and contain part of the att sequence. The universal external primers were used in the second PCR to complete the att sites. In the module-specific primers, sequence in capitals corresponds to the oligonucleotide segment that anneals to the template, while the sequence in bold type is annealed by the universal external primer that will complete the corresponding att site. The same forward and reverse primers were used for the PCR amplification of EGFP, ECFP and EYFP, since the mutations dictating the fluorescence wavelength lie beyond the sequence annealed by the primers. The N-terminal V5-6xHis module was PCR-amplified with a three-step PCR. The first forward module-specific primer (a) attached a Kozak sequence and an initiation methionine codon to the cassette containing the epitope tags, but no att-related sequence (∅), while the second PCR was carried out with a second forward primer (b) that provided the seed for the attB4 site. This site was completed in the last PCR, which was carried out with the corresponding external universal primers. Only one reverse module-specific primer was used in the first and second PCRs for this module. [file 1471-2199-14-18-S3.docx]

| **Module** | **Module position** | **att site** |  | **Sequence (5’→3’)** |
| --- | --- | --- | --- | --- |
| p65 | central | B1 | fw | **aaagcaggctcc**gccgccATGGACGAACTGTT |
|  |  | B2 | rv | **gtacaagaaagctgggtt**GGAGCTGATCTGACTCAG |
| ΔN-termSIRT1 | central | B1 | fw | **aaagcaggctcc**gccgccATGGCGGACGAGGTGGC |
|  |  | B2 | rv | **gtacaagaaagctgggtt**TGATTTGTCTGATGGATAG |
| PAR-2 | central | B1 | fw | **aaagcaggctcc**gccgccATGCGAAGTCTCAG |
|  |  | B2 | rv | **gtacaagaaagctgggtt**GTAGGAGGTTTTAACACT |
| mKate2 | N-term | B4 | fw | **aaagttgcc**gccaccATGGTGAGCGAGCTG |
|  |  | B1R | rv | **tttgtacaaacttgt**TCTAGATCCGGTGGAT |
| V5_6xHis | N-term | Ø | fw (a) | gccaccatgGGTACCGAGCTCGGATCC |
|  |  | B4 | fw (b) | **aaagttgcc**gccaccATGGGTACCGA |
|  |  | B1R | rv | **tttgtacaaacttgt**ATGGTGATGGTGATGAT |
| EGFP, ECFP, EYFP | N-term | B4 | fw | **aaagttgcc**accATGGTGAGCAAGGGCGA |
|  |  | B1R | rv | **tttgtacaaacttgt**GTACAGCTCGTCCATGCC |
| mKate2 | C-term | B2R | fw | **aaagtggac**ATGGTGAGCGAGCTGATTAA |
|  |  | B3 | rv | **gtataataaagttgt**TTATCTAGATCCGGTGGAT |
| V5_6xHis | C-term | B2R | fw | **aaagtggac**GGTACCGAGCTCGGATCC |
|  |  | B3 | rv | **gtataataaagttgt**TCAATGGTGATGGTGATGAT |
| EGFP, ECFP, EYFP | C-term | B2R | fw | **aaagtggac**ATGGTGAGCAAGGGCGA |
|  |  | B3 | rv | **gtataataaagttgt**TTAGTACAGCTCGTCCATGC |
| SV40 polyA | C-term | B2R | fw | **ttcttgtacaaagtggac**AACTTGTTTATTGCAGC |
|  |  | B3 | rv | **ctttgtataataaagttgt**CAGACATGATAAGATACA |
| IRES-ECFP | C-term | B2R | fw | **aaagtggac**ATTCTGCAGTCGACGGTA |
|  |  | B3 | rv | **gtataataaagttgt**TCAGTCAGGCGTGATGGTGG |

**Module-specific primers**

**Universal external primers**

| **Module position** | **att site** |  | **Sequence (5’→3’)** |
| --- | --- | --- | --- |
| Central | B1 | fw | ggggacaagtttgtacaaaaaagcaggctcc |
| Central | B2 | rv | ggggaccactttgtacaagaaagctgggtt |
| N-term | B4 | fw | ggggacaactttgtatagaaaagttgcc |
| N-term | B1R | rv | ggggactgcttttttgtacaaacttgt |
| C-term | B2R | fw | ggggacagctttcttgtacaaagtggac |
| C-term | B3 | rv | ggggacaactttgtataataaagttgt |
